# Host cytokine responses distinguish invasive from airway isolates of the *Streptococcus*milleri/anginosis group

**DOI:** 10.1186/1471-2334-14-498

**Published:** 2014-09-11

**Authors:** Julienne C Kaiser, Chris P Verschoor, Michael G Surette, Dawn ME Bowdish

**Affiliations:** Department of Biochemistry and Biomedical Sciences, McMaster University, 1280 Main St W, Hamilton, ON L8S 4 K1 Canada; Department of Pathology & Molecular Medicine, McMaster Immunology Research Centre, McMaster University, 1280 Main St W, Hamilton, ON L8S 4 K1 Canada; Department of Medicine, Farncombe Family Digestive Health Research Institute, McMaster University, 1280 Main St W, Hamilton, ON L8S 4 K1 Canada

**Keywords:** *Streptococcus* milleri group, Commensal, Cytokines, Host response, Peripheral blood mononuclear cells

## Abstract

**Background:**

The *Streptococcus* Milleri/Anginosus Group (SMG) colonize mucosal surfaces, especially the airways, and are considered to be normal mucosal microbiota; however, they are a major cause of abscesses, pneumonia and pleural empyema. The production of exoenzymes and virulence factors do not correlate with SMG pathogenicity. Since SMG infections are associated with robust inflammatory responses, we hypothesized that host immune responses might distinguish strains associated with asymptomatic carriage and those associated with fulminant disease.

**Methods:**

We measured IL1β, TNF, IL10, IL12, IL23, IL17, and IL4 production from human peripheral blood mononuclear cells (PBMCs) stimulated with a panel of clinical isolates from the airways and infections and measured the ability of these isolates to stimulate TLR2.

**Results:**

Isolates were categorized based on the levels of cytokines they induced from PBMCs (high, intermediate, low). Airway isolates predominantly induced low levels of cytokines and isolates from invasive disease induced higher levels, although about 10% of the strains produced divergent cytokine responses between donors. Interestingly, the donors were most divergent in their production of IL17, IL12 and IL23.

**Conclusions:**

We propose that the ability to inhibit or avoid an inflammatory response is associated with carriage in the airways and variability in responses between isolates and donors might contribute to susceptibility to disease.

**Electronic supplementary material:**

The online version of this article (doi:10.1186/1471-2334-14-498) contains supplementary material, which is available to authorized users.

## Background

The *Streptococcus* Milleri/Anginosus Group (SMG), including the three species *Streptococcus anginosus*, *Streptococcus constellatus*, and *Streptococcus intermedius*, are considered commensal bacteria since they asymptomatically colonize the gastrointestinal, female urogenital, and respiratory tracts of 15-30% of the healthy population [1, 2]. They are, however, also causative agents of pyogenic infections, accounting for 28-40% of brain abscesses [3, 4], 20-44% of liver abscesses [5–7], 20% of head and neck abscesses [8], and 30-50% of pleural empyema [9, 10], all of which are often complicated by bacteremia [11]. In addition to causing acute invasive infections, we have recently identified the SMG as a chronic respiratory pathogen in cystic fibrosis (CF) patients [12]. It is unclear whether there are separate disease-causing and commensal strains of the SMG. This is complicated largely by the vast genetic and phenotypic heterogeneity of the three species. Clinical isolates of the SMG exhibit inter- and intra-species diversity in phenotypic characteristics, including biochemical properties, Lancefield grouping (A, C, G, F, non-groupable), hemolysis on sheep’s blood agar (α, β, γ), and production of putative virulence factors including the hydrolytic enzymes hyaluronidase (HA), chondroitin sulfatase (CS), DNase, and proteases. Previous studies have shown that there is no association between the phenotype of an isolate and its ability to cause infection [13, 14].

Since studies of phenotypic characteristics have not been informative in distinguishing invasive or commensal isolates of the SMG, we reasoned that host immune responses might contribute to pathogenicity. The pathology of bacterial infections can be mediated either by damage to host tissues directly or by the host’s immune response. If the inflammatory response is not appropriately calibrated, it can drive tissue damage or, in systemic infections, excess cytokine production, which can be fatal [15, 16]. SMG infections are characterized by robust neutrophilia and local and systemic inflammation [17], whereas carriage (approximately ≤1 × 10^5^ CFU/mL in the lungs) does not appear to be accompanied by a robust immune response [18, 19].

Commensal bacteria might be less immunogenic than pathogenic bacteria or might induce an anti-inflammatory response that contributes to their selection as long-term residents of mucosal surfaces. For example, in a study comparing dendritic cell production of IL23, IL12p70 and IL10 after exposure to airway isolates, it was found that the pathogenic species *Morexella catarrhalis* and *Haemophilus influenzae* induced high levels of cytokines, non-pathogenic isolates of *Veillonella* spp. and *Actinomyces* spp. induced low levels of cytokines and non-pathogenic isolates of *Prevotella* spp. induced low levels of the inflammatory cytokines IL23 and IL12p70 but high levels of the anti-inflammatory cytokine IL10 [20]. Similarly, the commensal bacteria *Staphylococcus aureus* provokes divergent cytokine responses from macrophages, peripheral blood mononuclear cells (PBMCs), and dendritic cells that possibly account for the varying outcomes of carriage or infection. Macrophages, the sentinel cells of the nasopharynx where *S. aureus* is a common colonizing organism, produce IL10 in response to the bacteria, which is speculated to contribute to colonization. Dendritic cells, which are found in the circulation and in the skin, produce IL12 or IL23, which might contribute to the immunopathology of a systemic *S. aureus* infection [21–23]. These studies suggest that cytokine responses could be determinants of commensalism or pathogenicity.

To determine if cytokine responses to the SMG distinguish commensal from pathogenic isolates, we measured the production of IL1β, TNF, IL10, IL23, 12p70, IL17A, and IL4 from human PBMCs in response to 35 clinical isolates from either invasive infections (abscesses, bacteremia, empyema) or the CF airway cultured during periods of stability or exacerbation. Cytokine profiles differed across the 35 isolates resulting in a spectrum of responses that separated into high, intermediate, and low levels of cytokine production. The high and intermediate response groups were enriched for invasive isolates, which induced significantly higher levels of IL1β, TNF, IL10, IL23, and IL12p70 production than did airway isolates. The spectrum of cytokine profiles illustrates the heterogeneity of the SMG and indicates that cytokine responses might contribute to the potential of an isolate to colonize the airways or cause infection.

## Methods

### Bacterial strains

The 35 isolates of SMG used in this study included three ATCC reference strains (*S. anginosus* strain ATCC33397, *S. constellatus* strain ATCC27823, and *S. intermedius* strain ATCC27335) and 32 clinical isolates from a larger collection described previously [13]. Isolates from invasive infections (hip abscess, brain abscess, empyema, blood) were obtained from the Calgary Laboratory Services, Calgary, Canada. Airway isolates were cultured from the sputum of CF patients during periods of stability or exacerbation as part of standard patient care with ethical approval by the Conjoint Health Ethics Board of the Faculties of Medicine, Nursing and Kinesiology, University of Calgary, and the Affiliated Teaching Institutions of the Calgary Zone, Alberta Health Services. Isolates were considered associated with exacerbation when they were the most abundant microorganism cultured and this was always at an abundance from CF sputum at numbers ≥ 1 × 10^8^ CFU/mL. The SMG were grown in Todd Hewitt Broth containing 5 g/L yeast (THY) at 37°C, 5% CO_2_. Twenty-four hour cultures were centrifuged at 15,000 × g for 5 minutes, washed once with PBS and re-suspended in PBS. CFUs were enumerated by plating serial dilutions on THY agar followed by incubation at 37°C, 5% CO_2_ for 24 hours. Cell suspensions were heat-killed for 10 minutes at 65°C. Bacterial phenotypes were characterized and previously assigned to biotypes [13, 24]. Briefly, hydrolytic enzyme activity and hemolytic properties were determined by plating bacteria on differential agar. Lancefield groups were determined by a commercially available kit (Oxoid, Nepean, Ontario, Canada).

### Primary cell culture & PBMCs stimulation

Protocols were approved by the McMaster Research Ethics Board and written informed consent was obtained for all participants. Human PBMCs were isolated from the heparinized venous blood of healthy human donors (n = 3, age range 30–50 years, male:female 2:1) using Ficoll-Paque (GE Healthcare) and Leucosep tubes (Greiner Bio-One). Cells were cultured in X-VIVO 10 media (Lonza) containing gentamicin and 1% autologous plasma. Cells were seeded at a density of 5 × 10^5^ cells/well in a 96-well round bottom plate in a final volume of 200 μL. Heat-killed SMG were added to freshly isolated PBMCs in triplicate at a ratio of 5:1 (PBMCs:bacteria) in a final volume of 210 μL. After 24 hours of stimulation, PBMCs were centrifuged at 1,600 × g for 5 minutes and supernatants were collected and stored at −20°C until analyzed. The levels of IL1β, TNF, IL10, IL23, IL12p70, IL17A, and IL4 were measured in cell supernatants using the MILLIPLEX Map Human Th17 Magnetic Bead Panel (EMD Millipore) following the manufacturer’s recommendations on the Luminex xMAP platform. Release of lactate dehydrogenase (LDH) from PBMCs was used to assess PBMC viability using the CytoTox96 non-radioactive cytotoxicity assay kit (Promega) following the manufacturer’s instructions. The formula (OD of treated - OD of untreated)/(OD of complete lysis control - OD of untreated) × 100 was used to calculate percentage of non-viable cells.

### Analysis of cytokine responses

Cytokine data for each donor were scaled on a scale of 0 to 1 with highest value set to 1 to generate the heatmap and subsequent dendrogram for the cluster analyses, as well as for principle coordinate analysis (PCoA). Cluster analyses were performed by calculating the distance matrix using the Euclidean method followed by complete linkage hierarchal clustering using the gplots package in R version 2.15.2. PCoA was performed using the Bray-Curtis method in the vegan package in R. For all other cytokine analyses, data are the mean cytokine value across all three donors and were analyzed using non-parametric statistical tests, as indicated.

### TLR2 NF-κB:SEAP reporter assay

The HEK-Blue-2 cell line (InvivoGen PlasmoTest) stably expresses TLR2, CD14 and a secreted embryonic alkaline phosphatase (SEAP) reporter gene inducible by the transcription factors NF-κB and AP-1. These cells were cultured in complete DMEM (2 mM L-glutamine, 10% fetal bovine serum, 50 U/mL penicillin, 50 μg/mL streptomycin) with the addition of 350 μg/mL hygromycin, 5 μg/mL blasticidin, and 25 μg/mL zeocin. The cells were seeded into a 96-well flat bottom plate (BD Biosciences) at a density of 2 × 10^4^ cells/well in complete DMEM. After 24 hours, the medium was replaced with HEK-Blue Detection medium (InvivoGen) and the cells were treated with 1 × 10^6^ heat-killed SMG cells or 10-fold dilutions of Pam3CSK4 (InvivoGen) (highest final concentration of 0.1 μg/mL) as a positive control. Each sample was studied in triplicate. Twenty-four hours later, the absorbance was read at 630 nm using a BioTek Synergy H1 plate reader. Fold change was calculated relative to the unstimulated control.

## Results

### *S. anginosus*, *S. constellatus*, and *S. intermedius*elicit similar cytokine responses from human PBMCs

Thirty-five isolates of SMG were selected from our collection of characterized clinical isolates [13, 25], including 12 *S. anginosus*, 12 *S. intermedius*, and 11 *S. constellatus*. The isolates varied in phenotypic traits as well as in the clinical source of isolation to represent the phenotypic and pathogenic diversity of the SMG (Additional file 1: Table S1). PBMCs were stimulated with heat-killed SMG for 24 hours, after which the levels of TNF, IL1β, IL10, IL23, IL12p70, IL17A, and IL4 were measured in cell supernatants. Since the SMG have variable rates of growth in the tissue culture media isolates were heat-killed in order to eliminate any variability that would occur due to bacterial growth within the 24 hour stimulation.

**Figure 1:**
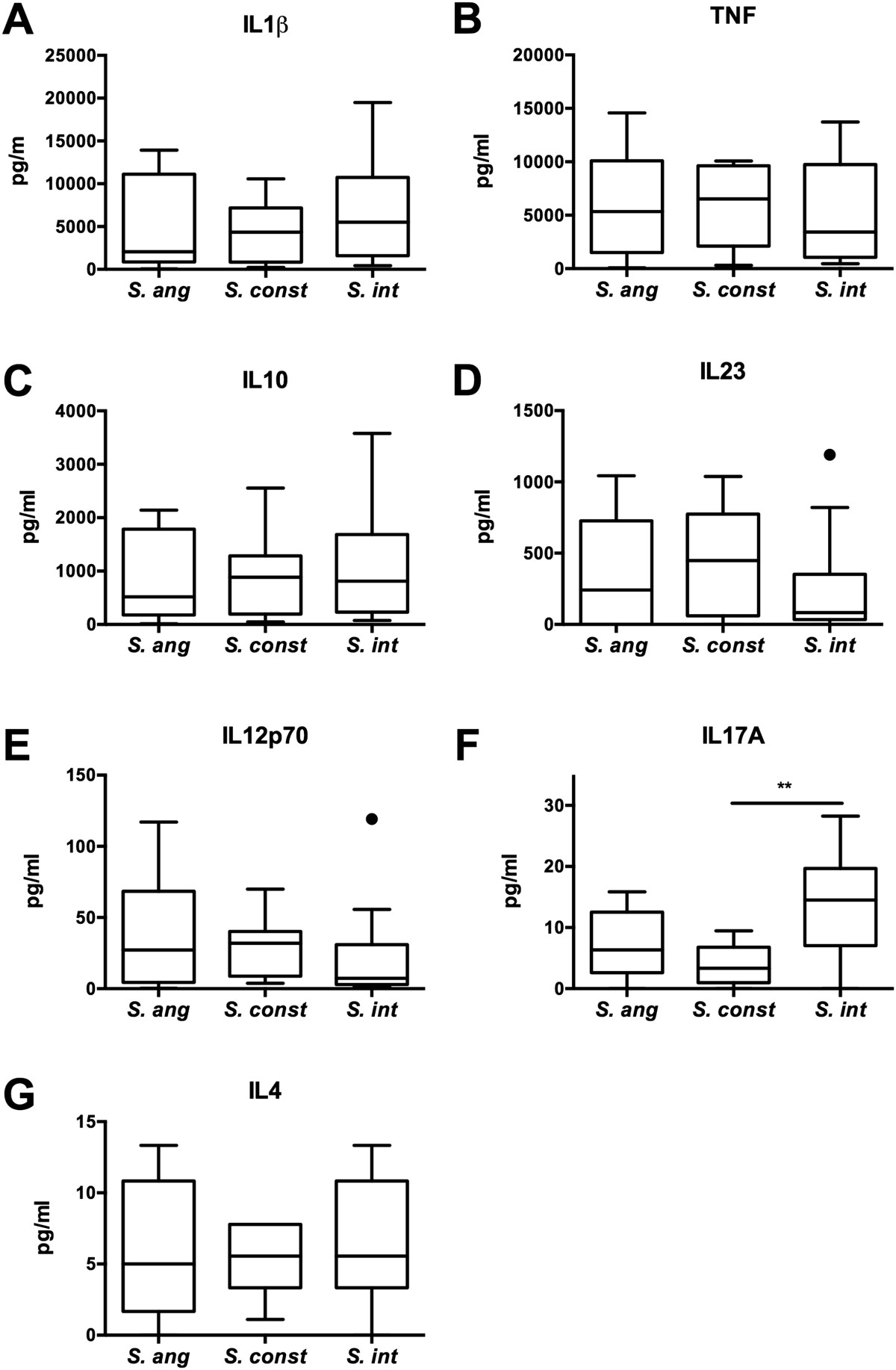
**Cytokine responses to clinical isolates of the SMG.** PBMCs were stimulated with 35 heat-killed isolates of the SMG (12 *S. anginosus*, 11 *S. constellatus*, and 12 *S. intermedius*) at a ratio of 5:1 (PBMCs:bacteria). Levels of IL1β, TNF, IL10, IL23, 12p70, IL17A and IL4 were measured in cell supernatant after 24 hours by multiplex assaying. **A-G**. Box-and-whisker plots indicate median, interquartile ranges and outliers of cytokine production in response to isolates of each species. *S. ang* = *S. anginosus*, *S. const* = *S. constellatus*, *S. int* = *S. intermedius*. Statistical significance was determined by Kruskal-Wallis test with Dunn’s post hoc comparison. ** *P* < 0.01.

**Figure 2:**
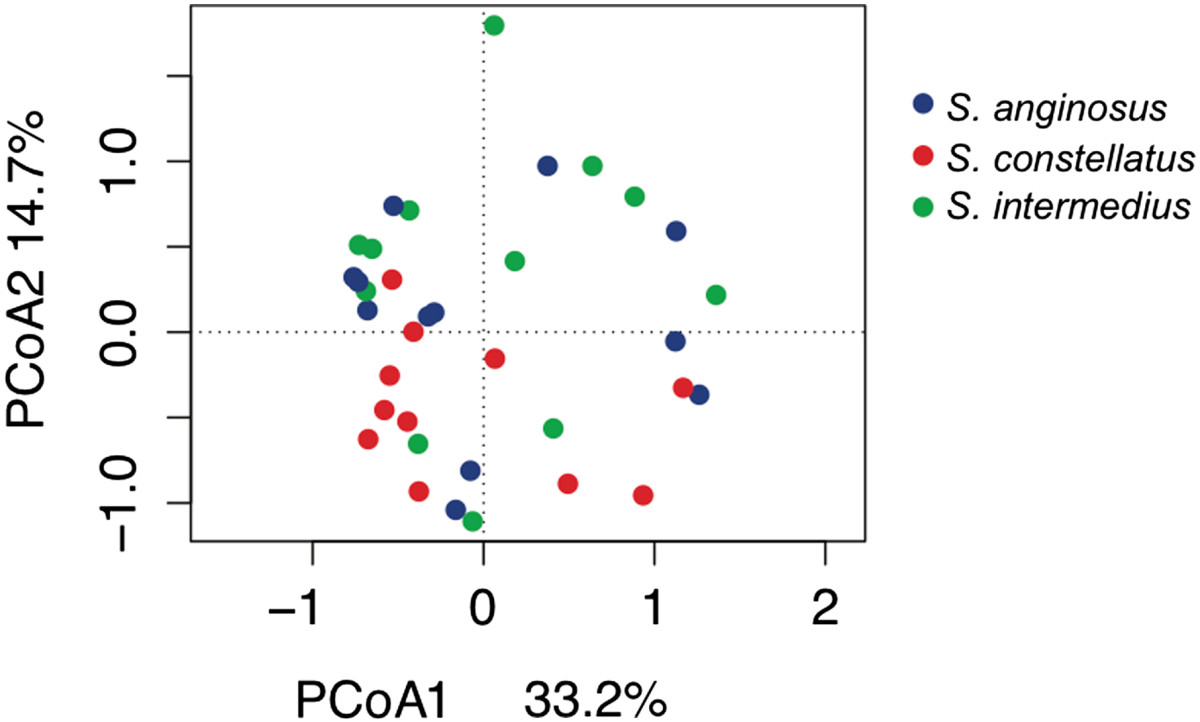
**The SMG do not induce species-specific cytokine profiles.** PCoA analysis of scaled cytokine profiles for all 35 isolates. Isolates are coloured by species.

The SMG induced a range of responses for each cytokine tested. Overall, the SMG induced amounts of IL1β, TNF, IL10, and IL23 10-1000-fold higher than IL12p70, IL17A and IL4. We compared cytokine levels across species to determine whether species classification explained the range of responses. Within a species, isolates varied in their ability to induce cytokine production from PBMCs, the median of which was comparable across the three species except for IL17A (Figure 1A-G). *S. intermedius* induced significantly higher IL17A production than *S. constellatus* (Figure 1F). Aside from this, the data suggest that the three species elicit similar cytokine responses. PCoA confirmed that the variability in cytokine production was not species specific (Figure 2).

### Cytokine profiles define a spectrum of cytokine responses to the SMG

**Figure 3:**
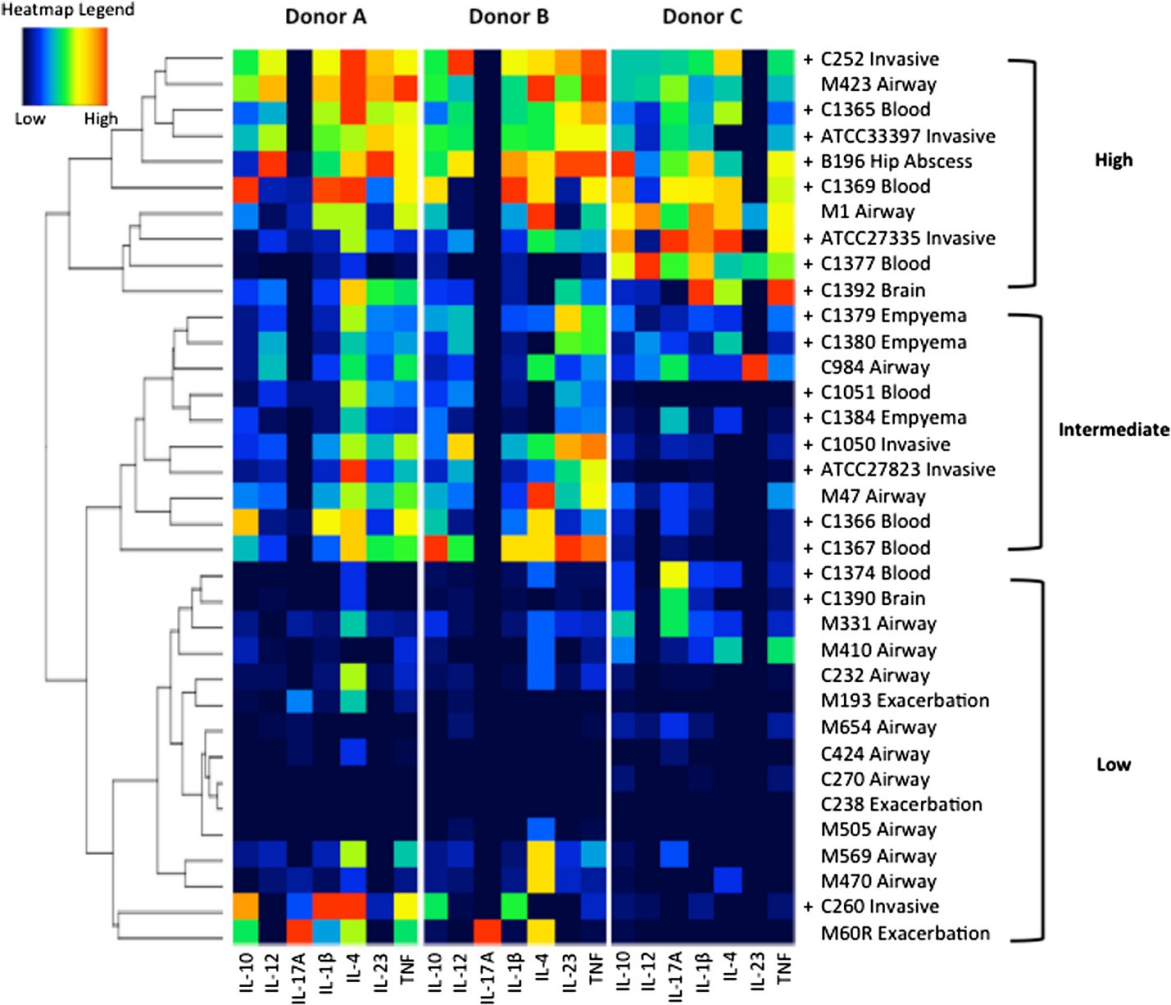
**Heatmap and cluster analysis of cytokine profiles for 35 isolates of the SMG.** Heatmap colours indicate scaled cytokine data for donors A, B, and C. Hierarchal clustering was used to generate the cluster dendrogram and cytokine response groups (high, intermediate, low) were assigned based on the branching of the dendrogram. Isolates are labeled with the isolate ID and clinical source. + indicates isolates originating from invasive infections. Heatmap legend: low = scaled value of 0, high = scaled value of 1.

**Figure 4:**
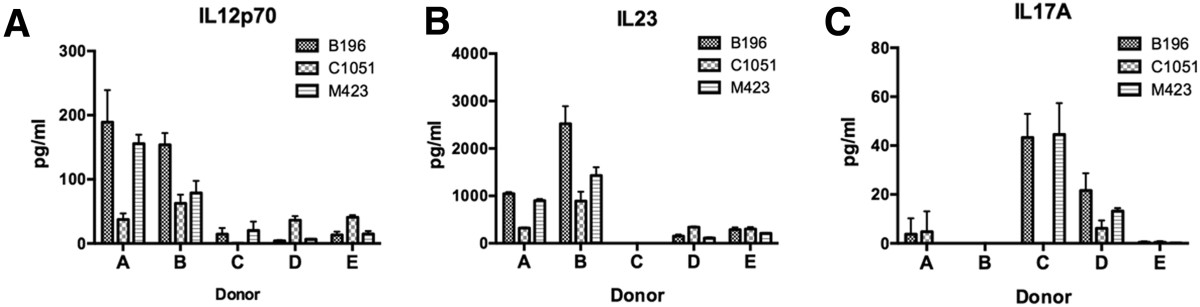
**Reciprocal IL-12/IL-23 and IL17A donor responses to the SMG.** PBMCs were stimulated heat-killed SMG including the *S.intermedius* isolate B196 and *S. anginosus* isolates C1051 and M423 at a ratio of 5:1 (PBMCs:bacteria). PBMC supernatants were collected after 24 h and IL12p70, IL23, and IL17A were measured by multiplex assaying. **A**. ILp1270 responses, **B**. IL23 responses, and **C**. IL17A responses for all 5 donors are shown. Data are mean of triplicates +/− SD.

We next generated a heatmap of the cytokine profiles for the 35 isolates from the three individuals and analyzed them by hierarchal clustering to visualize isolate-specific and donor-specific responses. The cluster analysis revealed three groups of cytokine profiles: high, intermediate, and low responses (Figure 3). Isolates did not cluster by species, corresponding with the PCoA results. Rather, the isolates separated based on whether they originated from invasive infections or the airway. In both the high and intermediate response groups, 80% of isolates originated from invasive infections, compared to 20% in the low response group of which the majority originated from the airways. Cytokine profiles did not group invasive isolates from the same source of infection (e.g. blood, brain) together. To eliminate the possibility that the lack of cytokine production for some isolates was due to cytotoxicity, the amount of LDH released by PBMCs following stimulation with the heat-killed isolates was measured. The SMG induced a range of cell death (1-20% cytotoxicity), however, cytotoxicity did not correlate with cytokine responses (data not shown). Thus, the spectrum of cytokine responses observed is due to differences in immunostimulatory properties of SMG isolates and not cell cytotoxicity.The placement of isolates within the cytokine spectrum and grouping of responses was consistent across the three donors with the exception of a few isolates. The isolates ATCC27335 and C1392 induced low cytokine production in donors A and B, yet induced a high cytokine response in donor C. Conversely, C260 and M60R induced low cytokine responses in donor C, yet high cytokine responses in donors A and B. Interestingly, donor’s A and B responses to C260 and M60R could constitute a separate response group, as they are the only isolates to induce an IL17A response in these donors. In addition to these differences in donor responses to select strains, we observed an overall divergence between donors in the production of IL23 and IL17A. In general, donor C produced IL17A in response to the majority of the immunostimulatory isolates but did not produce IL23 and produced relatively low levels of IL12p70. In contrast, donors A and B produced high levels of IL23 but no IL17A. Although the tendency of a donor’s response to skew towards either IL17A or IL23 held true for the majority of the isolates, the M60R isolate induced IL17A production in donors A and B and isolate C984 induced IL23 production in donor C, indicating that these responses are inducible. We investigated whether this donor-specific inverse relationship between IL23 and IL17A production also occurred in two additional donors. Three of the five total donors tested produced IL23 and IL12p70 but no IL17A to isolates B196, C1051, and M423 (donors A, B, and E) and the remaining two produced IL17A but little or no IL23 and IL12p70 to the same isolates (donors C and D) (Figure 4A-C). These data indicate that human variability contributes to the cytokines induced in response to the SMG.

### Invasive isolates induce higher IL-1β, TNF, IL-10, IL23, and IL12p70 production than airway isolates

**Figure 5:**
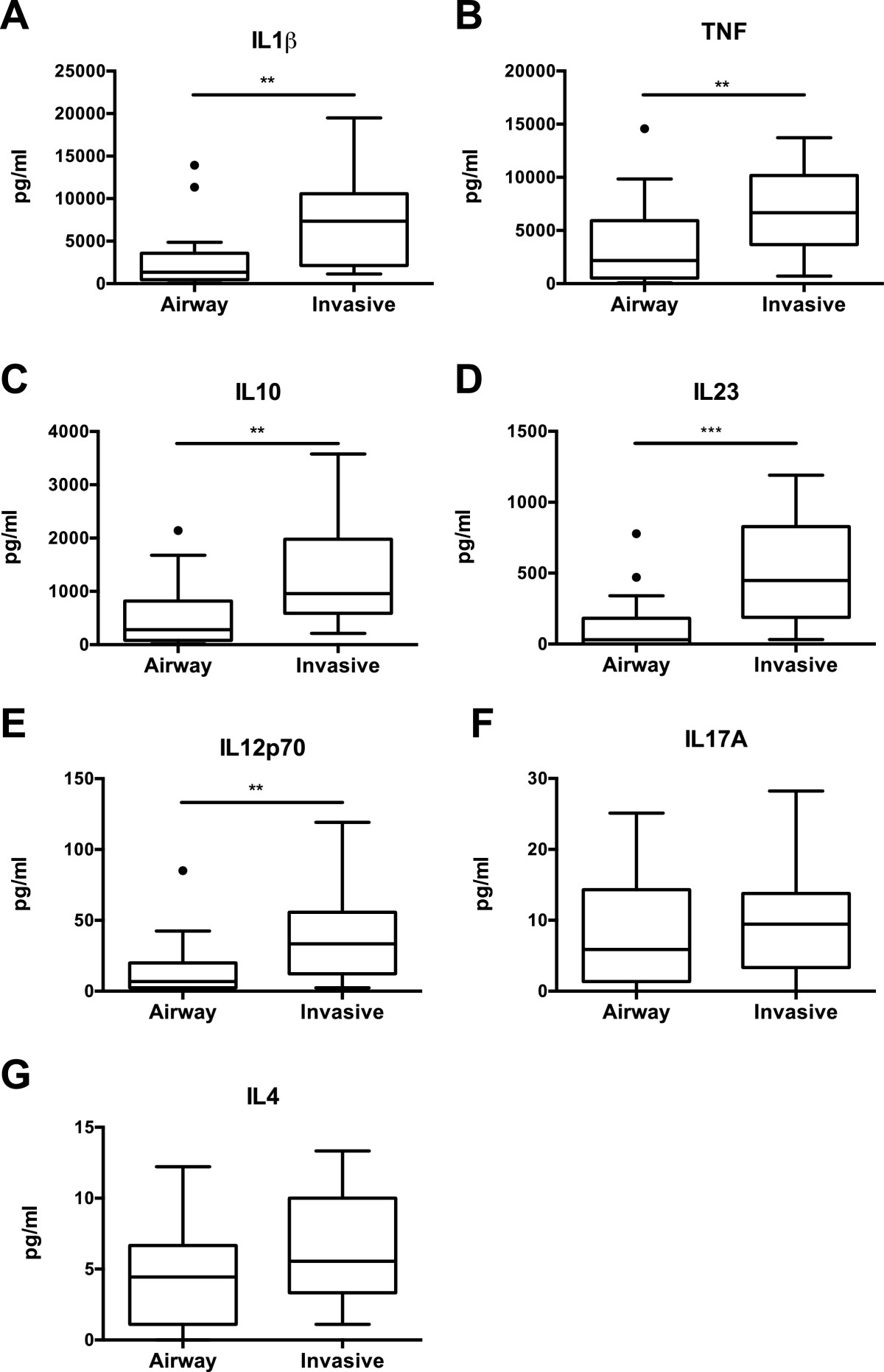
**Induction of IL1β, TNF, IL10, IL23, IL12p70, IL17A, IL4 by invasive and airway isolates of the SMG.** PBMCs were stimulated with 35 heat-killed isolates of the SMG from invasive infections (n = 19) or the airways (n =16). Levels of **A**. IL1β, **B**. TNF, **C.** IL10, **D**. IL23, **E**. 12p70, **F**. IL17A and **G**. IL4 were measured in cell supernatant after 24 hours by multiplex assaying. Box-and-whisker plots indicate median, interquartile ranges and outliers of cytokine production. Statistical significance between invasive and airway isolates was determined by a Mann–Whitney test. *P < 0.05, **P < 0.01.

We next compared whether the variability in cytokine responses was related to the source of isolation. Isolates from acute invasive infections (blood, brain, empyema, hip abscess) were compared to isolates from chronically colonized CF airways. Invasive isolates induced significantly higher production of IL1β, TNF, IL10, IL23, and IL12p70 from PBMCs than airway isolates (Figure 5A-E), but comparable levels of IL17A and IL4 (Figure 5F, G). Within the invasive isolates the cytokine profiles did not segregate based on infection site. These data suggest that cytokine production, including both pro- and anti-inflammatory cytokines, in general is an important feature of SMG infections, however the type of infection is not associated with a specific set of cytokines.

### Immunostimulatory properties of the SMG do not correlate with their phenotypic traits

Phenotypic traits, including Lancefield grouping, β-hemolytic activity on sheep’s blood agar, and production of protease, DNase, HA, and CS are used clinically to differentiate strains of the SMG [13, 24, 26]. Comparison of the phenotypic traits of isolates, as previously characterized by Grinwis et al.*,*[13] to cytokine production revealed no association between phenotypic traits and induction of TNF, IL1β, IL10, IL23 or IL4 (Table 1), except for, CS positive isolates, which induced significantly higher amounts of IL-17A than CS negative isolates (Table 1). Grinwis et al.*,* also identified phenotypic “biotypes” within each species. Biotypes are groups of isolates with shared phenotypic properties based on the results of 18 biochemical tests [13]. *S. anginosus* isolates were grouped as phenotypically active, phenotypically non-active and HA positive, and *S. constellatus* isolates were grouped as non-groupable/Lancefield group F non-active, Lancefield group F active, and Lancefield group C and β-hemolytic. It is suggested that certain biotypes might be more clinically relevant [13]; therefore we assessed whether a correlation exists between cytokine profiles and the biotypes of the isolates. *S. anginosus* isolates of the active biotype and *S. constellatus* isolates of the Lancefield Group F active biotype were present in only the intermediate response group, whereas the other biotypes were present in two or more response groups (Table 2). Therefore, the phenotypic trait or biotype of a clinical isolate does not enable identification of isolates with increased immunostimulatory properties.

**Table 1:** **Comparison of average cytokine induction by isolates based on phenotypic characteristics**

Phenotype	No. of isolates	IL1β		TNF		IL10		IL12p70		IL23		IL17A		IL4	
		pg/ml	***P***value	pg/ml	***P***value	pg/ml	***P***value	pg/ml	***P***value	pg/ml	***P***value	pg/ml	***P***value	pg/ml	***P***value
**Hyalurdonidase**															
Positive	26	5518	ns	5229	ns	916	ns	23	ns	302	ns	9	ns	6	ns
Negative	9	5089		6904		945		48		448		8		6	
**Chondroitan Sulfatase**															
Positive	15	6099	ns	4938	ns	968	ns	23	ns	267	ns	12	0.0337	6	ns
Negative	20	4889		6201		948		33		394		6		6	
**β-Hemolysis**															
Positive	16	4892	ns	5739	ns	916	ns	32	ns	380	ns	6	ns	6	ns
Negative	19	5843		5593		996		27		306		11		6	
**Protease**															
Positive	29	5704	ns	5491	ns	889	ns	29	ns	312	ns	9	ns	6	ns
Negative	6	3976		6477		1298		29		475		4		6	

*P* value determined by a Mann–Whitney test. *P* < 0.05 was considered significant.

**Table 2:** **Proportion of** ***S. anginosus*** **and** ***S. constellatus*** **biotypes in cytokine response groups**

Biotype	Total no. of isolates	Cytokine response group		
		Low	Intermediate	High
***S. anginosus***				
Active	3	0	3	0
Non-Active	6	3	0	3
HA Positive	3	2	0	1
***S. constellatus***				
Lancefield Group C, β-hemolytic	3	2	1	0
Lancefield Group F Active	2	0	2	0
NG/Lancefield group F non-active	6	1	4	1

*HA* hyaluronidase, *NG* non-groupable.

### Immunostimulatory properties do not correlate with TLR2 activation

**Figure 6:**
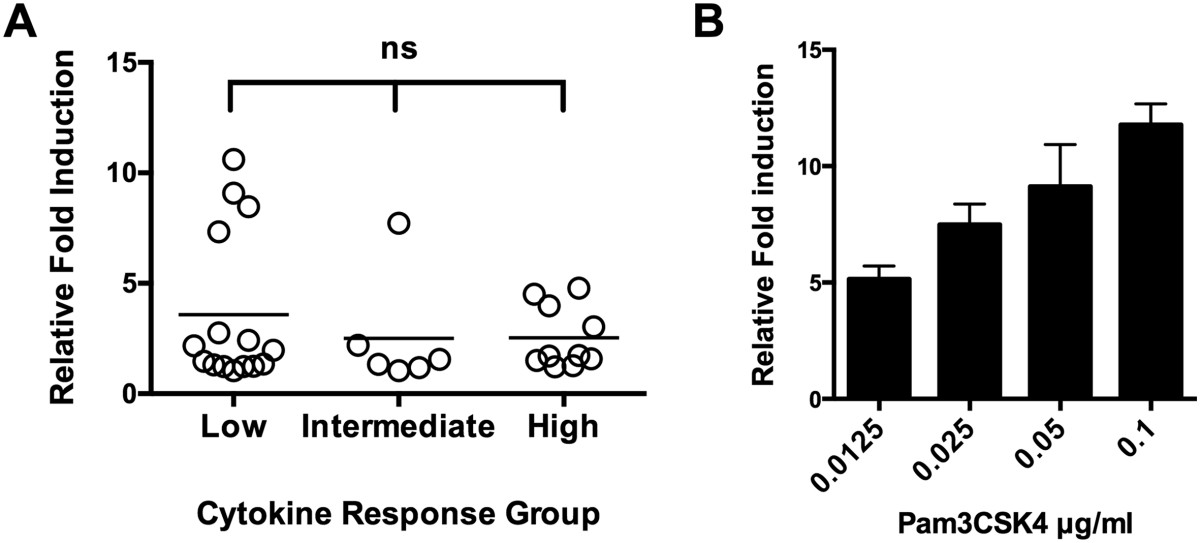
**Activation of TLR2 in response to the SMG. A**. HEK293 cells expressing TLR2 and an NF-κB:SEAP reporter were stimulated with heat-killed SMG from the low (n = 15), intermediate (n = 6), or high (n = 10) cytokine response groups at a ratio of 500:1 bacteria:HEK293 cells. Detection of SEAP expression was determined by measuring absorbance at 630 nm after 24 hours. Circles represent the relative TLR2 induction by a single isolate. Data are the mean of three replicates. Statistical significance was determined by Kruskal-Wallis test with Dunn’s post hoc comparison. Ns, not significant. **B**. Reporter cells were stimulated with serial dilutions of the TLR2 ligand Pam3CK4 for 24 hrs and OD_630_ was measured.

TLR2 is one of the primary pattern recognition receptors involved in recognition of Gram-positive bacteria and has been demonstrated to have divergent signaling pathways that contribute to either the establishment of commensalism or infection [27–29]. We examined whether the SMG utilize TLR2 using a HEK293 NF-kB:SEAP reporter cell line expressing TLR2 and CD14. The SMG induced a range of TLR2 induction (1-11-fold increase relative to unstimulated control) (Figure 6A) comparable to the TLR2 ligand Pam3CSK4 (Figure 6B). Comparison of TLR2 induction by isolates based on their location in the cytokine spectrum (low, intermediate, or high group) revealed no significant difference and unexpectedly, some isolates from the low cytokine response group activated TLR2 signaling in this system as well or better than isolates from the high cytokine response group (Figure 6A). Evidently, TLR2 activation alone does not explain the spectrum of cytokine responses to the SMG.

## Discussion

The SMG are a phenotypically and genetically heterogeneous group of bacteria that are members of the mucosal microbiota yet are frequent and serious pathogens. To date, no virulence factors have been identified that are associated with isolates originating from invasive infections, and infections do not appear to be the result of clonal expansion of a hypervirulent strain [13, 14, 19]. In this study, we investigated whether immune recognition of the SMG distinguishes isolates of commensal or disease origin. We demonstrated that there exists intra-species variation in cytokine responses to the SMG, resulting in a spectrum that ranges from high induction of both pro- and anti-inflammatory cytokines, to little or no induction of these cytokines. Within this spectrum, the majority of isolates that induced the higher responses were of invasive origin. We hypothesize that these responses contribute to whether the host recognizes the SMG as commensals or pathogens.

No significant difference between the levels of cytokines produced by *S. anginosus, S. intermedius* or *S. constellatus* was observed, except for IL17A (Figure 1F). *S. intermedius* induced significantly higher amounts of IL17A compared to *S. constellatus* and a similar trend also exists between *S. intermedius* and *S. anginosus*. Th17 cells are considered the main IL17-producing cells of the adaptive immune system, and whether this IL17 is produced from pre-existing Th17 cell or innate immune cells such as γδ T cells, invariant natural killer T cells, natural killer cells, and myeloid cells is not clear (reviewed in [30]). Ongoing studies are aimed at determining the cell type required for IL17 production, and identifying the ligands recognized. Aside from this difference, the similarity in cytokine responses across species suggests that immune recognition occurs via a common surface antigen. Moreover, we did not observe an association between phenotypic profiles and immunostimulatory properties (Table 1), which is consistent with data demonstrating that pathogenicity does not correlate with *in vitro* phenotypic properties such as exoenzyme production [13, 14]. The clinical source of the isolate, whether of commensal or invasive origin, could, however, explain the differences in cytokine production (Figure 5). This could be due to differential expression of cell surface ligands, however, the SMG are naturally competent [31] and analysis supports genomic diversity [32]. Therefore, additional genetic factors that result in variation of cell-surface molecule expression could contribute to the heterogeneity in immune recognition. Interestingly, although levels of cytokine production as a whole did not correlate with the phenotypic properties of the SMG, CS positive isolates induced elevated levels of IL17A production (Table 1). Whether this is causal or correlates with other surface properties of these isolates warrants further investigation.

Although the mechanisms of SMG pathogenicity are not well understood, it is well documented that the production of pro-inflammatory cytokines, especially IL1β and TNF are involved in both driving and preventing pyogenic infections [33, 34]. Whether the induction of IL1β and TNF by the SMG is protective or detrimental to the host is yet to be determined but it does appear to be a common characteristic of invasive isolates.

We included IL23, IL12p70, and IL4 in the cytokine panel because of their role in the polarization of CD4+ cells towards specific T helper (Th) cell lineages and there are no data on the type of Th cell population generated in response to the SMG. The SMG induced higher levels of IL23 than IL12p70 and IL4 (average of 12-fold induction of IL23 relative to IL12 and 66-fold induction relative to IL4). IL23 contributes to development of human Th17 cells [35, 36] and enhances IL17 production from stimulated T cells [37]. There is an increasing amount of evidence to support a role for Th17 cells and IL17 in the control of colonization of respiratory pathogens and progression to invasive infections, including *Streptococcus pneumoniae*[38, 39], *Klebsiella pneumoniae*[40, 41] and *Candida albicans*[42–44]. Future studies will address whether the SMG-induced IL23 production contributes to polarization of Th17 cells. Moreover, the donor differences in IL12/23 and IL17A production (Figure 4) could result in divergent downstream immune responses. IL17 is a potent mediator of neutrophil chemotaxis [45, 46], and therefore differences in IL17 production might contribute to the susceptibility to immunopathology of pyogenic infections.

Since it has previously been demonstrated that residents of the airways tend to induce IL10 production that promotes their colonization but might ultimately impair a protective immune response during systemic infection [47, 48], we hypothesized that airway isolates might induce IL10 and not the pro-inflammatory cytokines TNF or IL1β. This, however, was not the case. Invasive isolates produced the highest levels of IL10, and production of IL10 appeared to be co-regulated with IL1β and TNF (Additional file 2: Figure S1). In contrast, the airway isolates induced low levels of IL10 and pro-inflammatory cytokines in general. Since the isolates were heat-killed and our pilot experiments demonstrated that the supernatants did not induce a strong inflammatory response (data not shown), we hypothesized that the differences in cytokine responses are related to pattern-recognition signaling in response to ligands expressed on the surface. Since TLR2 is the dominant pattern recognition receptor involved in recognition of surface components of Gram-positive bacteria we used a TLR2-NF-κB reporter assay to test this hypothesis; however, we did not observe a correlation between TLR2 activation and cytokine production (Figure 6A). In fact, some isolates in the low cytokine group induced the highest TLR2 activation (Figure 6A). Additional co-receptors or alternative pattern recognition receptors on PBMCs presumably influence cytokine production.

The overall low immunostimulatory capacity of airway isolates could be the result of a selective pressure for minimally immunostimulatory isolates in the airways since the inflammation that would result from a response to more immunostimulatory isolates could compromise lung function [49]. Therefore, immunostimulatory isolates would be removed during this dynamic process of immunological homeostasis. Isolates M505 and M331, both of which were non-immunostimulatory isolates, were cultured on multiple occasions from the CF lung, supporting the notion that non-immunostimulatory isolates are selected as chronic colonizers in the airway [19]. We did, however, observe several airway isolates in the high and intermediate response group, including M423, M1, C984, and M47, suggesting that there might be periodic carriage of immunostimulatory isolates.

Despite the small the number of donors used for the PBMC studies, we observed an isolate-dependent cytokine response in all donors tested, supporting that such responses could determine whether an isolate is perceived as a commensal or a pathogen in a certain individual.

## Conclusions

Clinical isolates of the SMG exhibited a great degree of intra-species variation in induction of pro- and anti-inflammatory cytokines from human PBMCs, the magnitude of which correlated with whether the isolate originated from an invasive infection or colonization of the airways. Consequently, isolate diversity dictates host-responses and could ultimately result in asymptomatic carriage or, given the right conditions (e.g. co-morbidities, surgery or trauma, co-infection) symptomatic disease.

## Electronic supplementary material

Additional file 1: Table S1: Clinical source of the SMG isolates used in this study. (DOCX 22 KB)

Additional file 2: Figure S1: IL10 production is positively correlated with TNF and IL1β production. Levels of IL1β, TNF, and IL10 for all 35 isolates were plotted as pairwise plots for each donor to demonstrate a positive correlation between A-C. IL1β vs. IL10 and D-F. TNF vs. IL10. The best-fit line and 95% confidence intervals are plotted. The r^2^ value was determined by linear regression analysis. (TIFF 314 KB)

Below are the links to the authors’ original submitted files for images.

Authors’ original file for figure 1

Authors’ original file for figure 2

Authors’ original file for figure 3

Authors’ original file for figure 4

Authors’ original file for figure 5

Authors’ original file for figure 6

## Abbreviations

ATCC: American Typed Culture Collection
CF: Cystic fibrosis
CFU: Colony forming unit
CS: Chondroitin sulfatase
DMEM: Dulbecco’s Modified Eagle Medium
DNase: Deoxyribonuclease
HA: Hyaluronidase
HEK: Human embryonic kidney
IL: Interleukin
LDH: Lactate dehydrogenase
PBMC: Peripheral blood mononuclear cell
PBS: Phosphate buffered saline
PCoA: Principle coordinate analysis
SEAP: Secreted embryonic alkaline phosphatase
SMG: *Streptococcus* Milleri/Anginosus group
THY: Todd Hewitt broth
TLR: Toll-like receptor
TNF: Tumor necrosis factor.
