# Supplementary material for: Host cytokine responses distinguish invasive from airway isolates of the Streptococcusmilleri/anginosis group
Source: BMC Infect Dis. 2014 Sep 11;14:498. doi: 10.1186/1471-2334-14-498 (PMC4175566; doi:10.1186/1471-2334-14-498)
Supplement: Supplementary file 1 — Additional file 1: Table S1: Clinical source of the SMG isolates used in this study. (DOCX 22 KB) [file 12879_2013_3803_MOESM1_ESM.docx]

**Additional file 1: Table S1. Clinical source of the SMG isolates used in this study.**

| Species | Clinical Source | No. of Isolates |
| --- | --- | --- |
| *S. anginosus* | Airway  Blood  Empyema  Exacerbation  Invasive^a^ | 7  1  1  1  2 |
| *S. constellatus* | Airway  Blood  Brain  Empyema  Exacerbation  Invasive^a^ | 3  2  1  2  1  2 |
| *S. intermedius* | Airway  Blood  Brain  Exacerbation  Hip abscess  Invasive^a^ | 3  4  1  1  1  2 |

^a^Source of infection unknown.
