# Supplementary figures and images for: Host cytokine responses distinguish invasive from airway isolates of the Streptococcusmilleri/anginosis group
Source: BMC Infect Dis. 2014 Sep 11;14:498. doi: 10.1186/1471-2334-14-498 (PMC4175566; doi:10.1186/1471-2334-14-498)

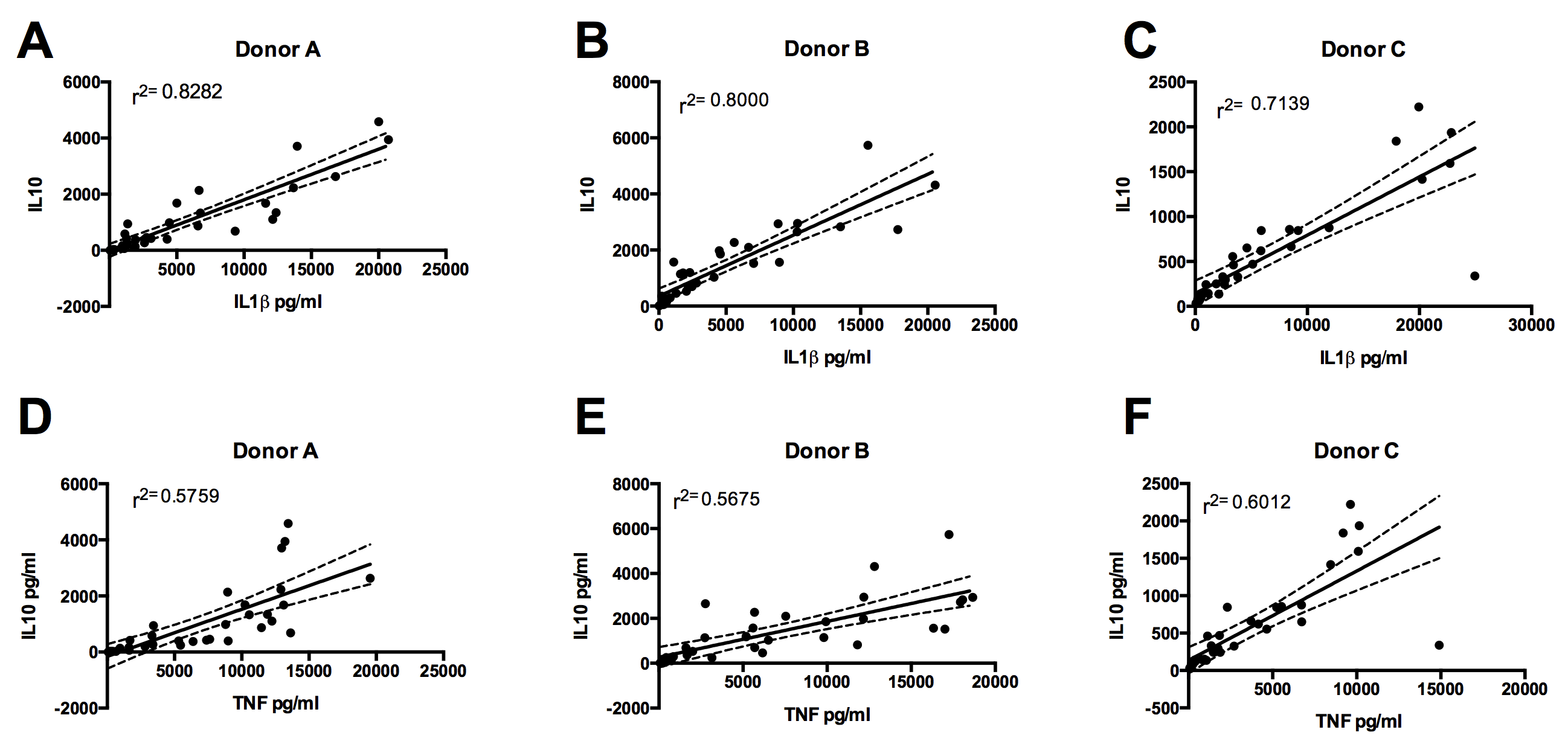

Supplement: Supplementary file 2 — Additional file 2: Figure S1: IL10 production is positively correlated with TNF and IL1β production. Levels of IL1β, TNF, and IL10 for all 35 isolates were plotted as pairwise plots for each donor to demonstrate a positive correlation between A-C. IL1β vs. IL10 and D-F. TNF vs. IL10. The best-fit line and 95% confidence intervals are plotted. The r2 value was determined by linear regression analysis. (TIFF 314 KB) [file 12879_2013_3803_MOESM2_ESM.tiff]

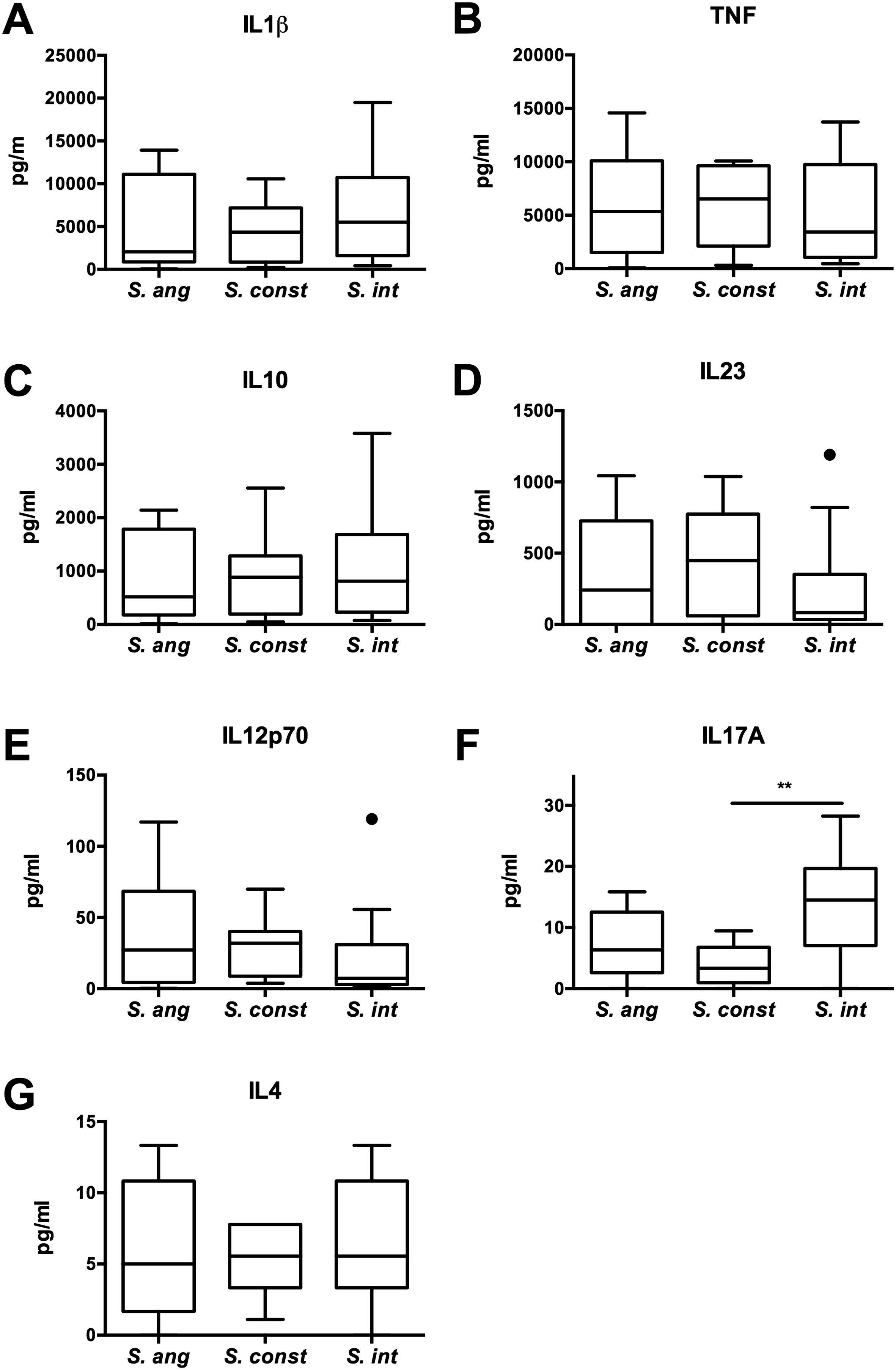

Supplement: Supplementary file 3 — Authors’ original file for figure 1 [file 12879_2013_3803_MOESM3_ESM.tif]

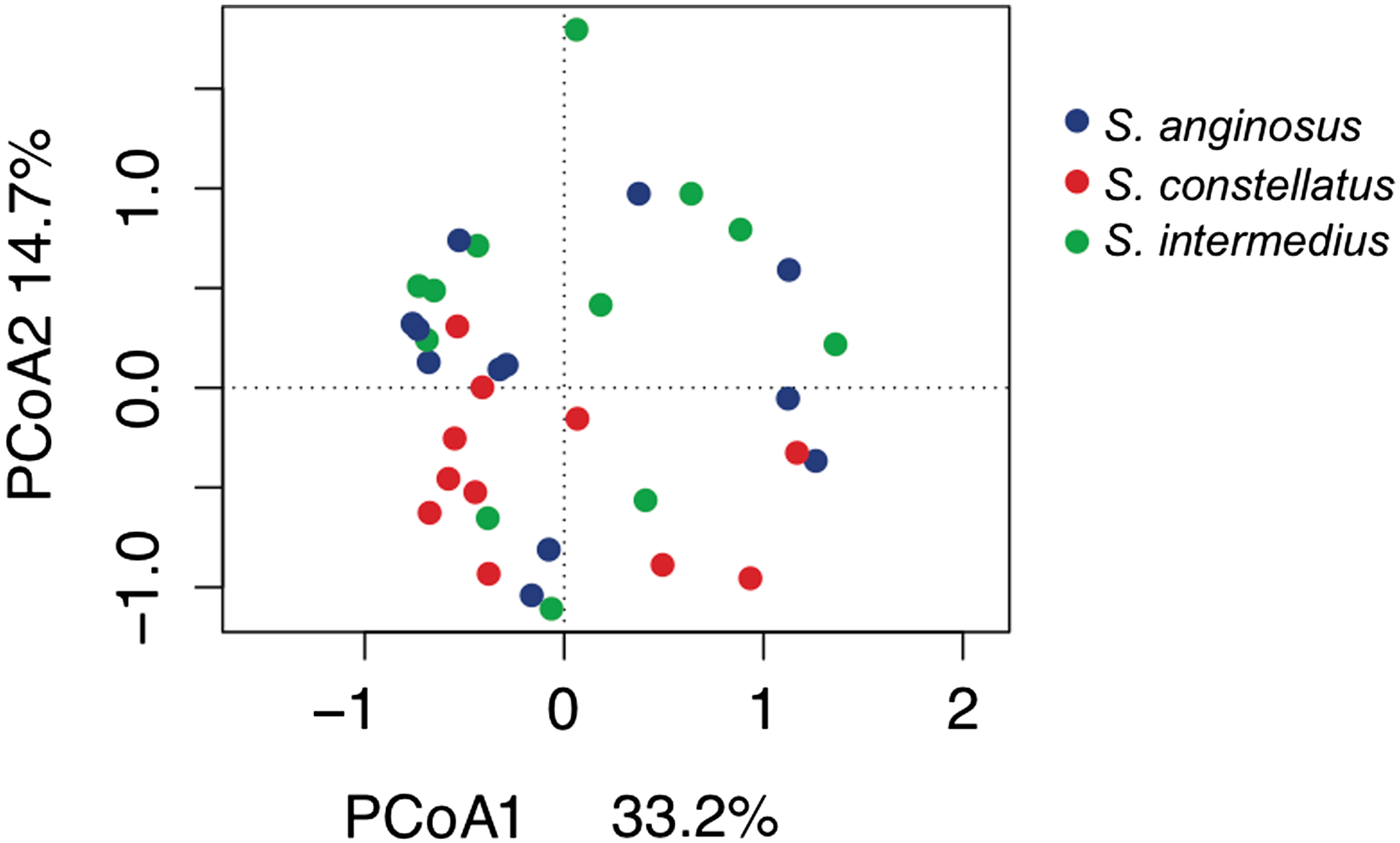

Supplement: Supplementary file 4 — Authors’ original file for figure 2 [file 12879_2013_3803_MOESM4_ESM.tiff]

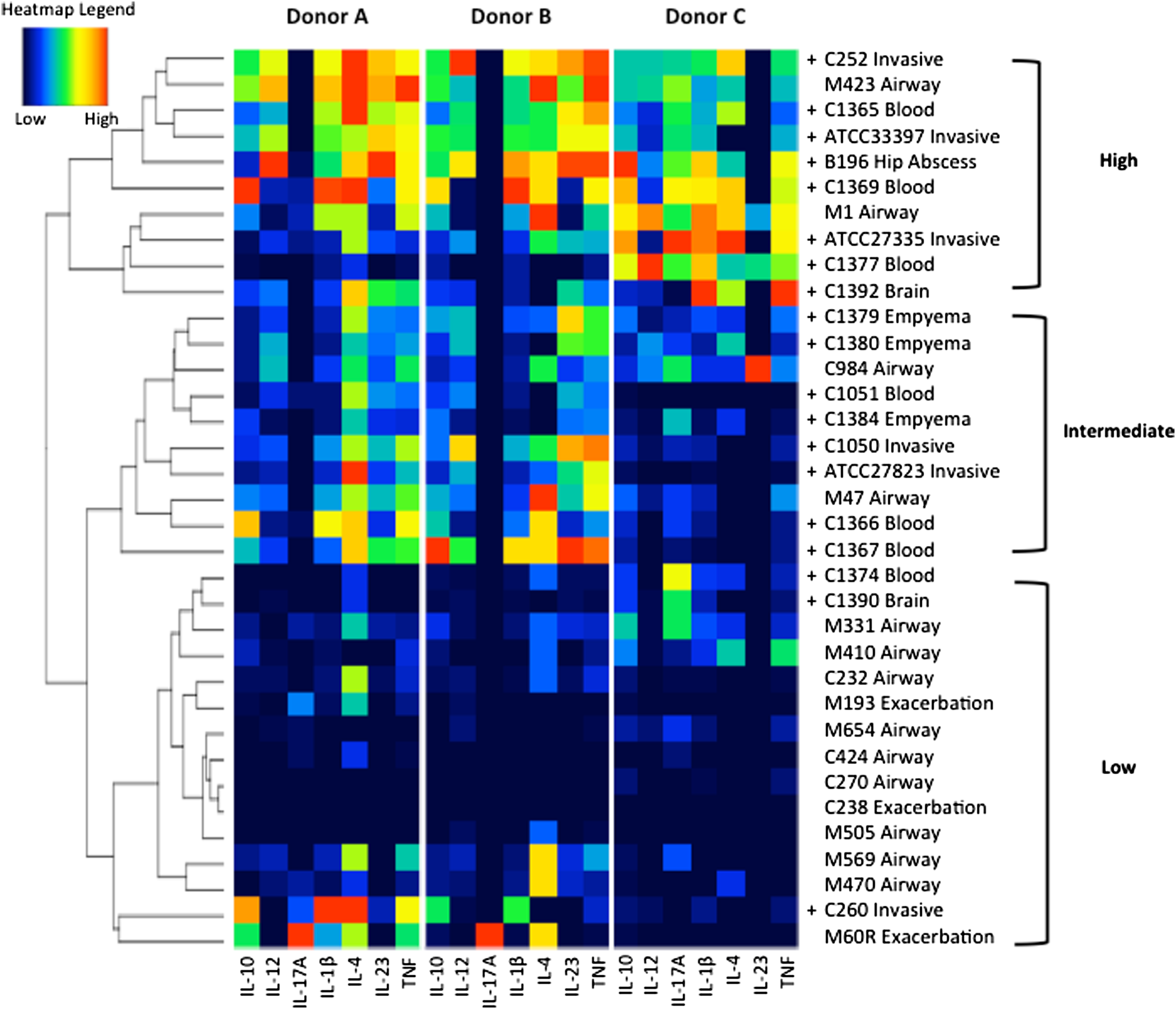

Supplement: Supplementary file 5 — Authors’ original file for figure 3 [file 12879_2013_3803_MOESM5_ESM.tif]

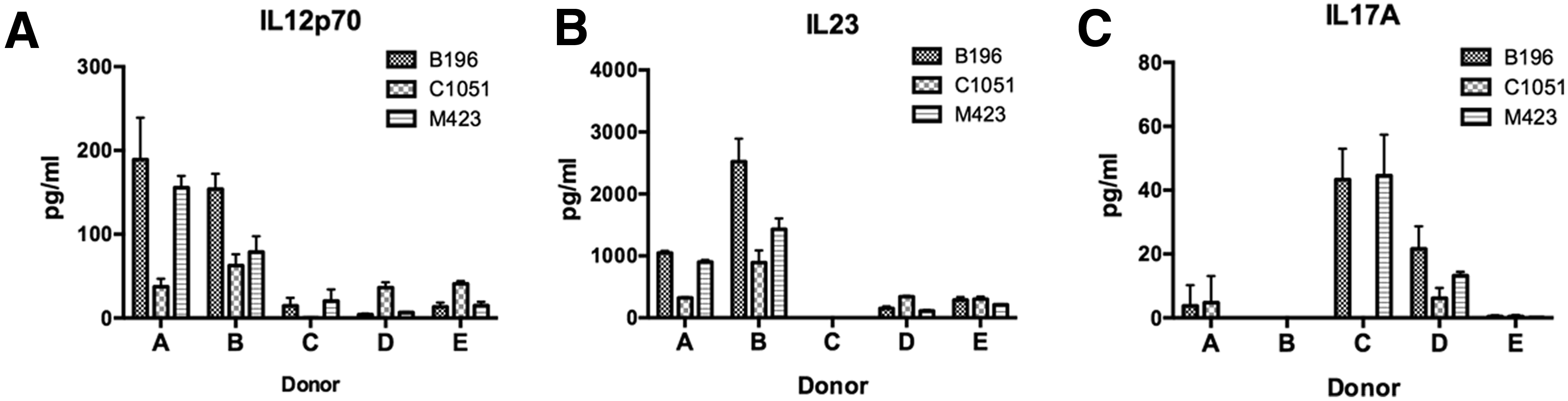

Supplement: Supplementary file 6 — Authors’ original file for figure 4 [file 12879_2013_3803_MOESM6_ESM.tif]

**A**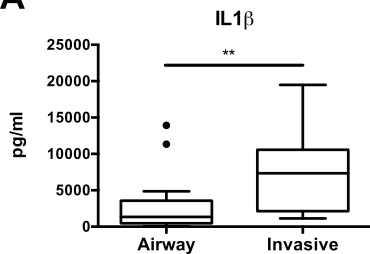**B**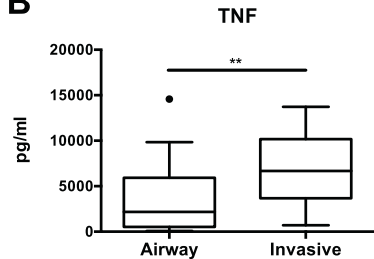**C**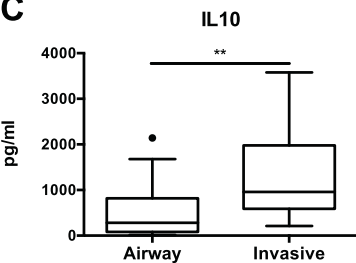**D**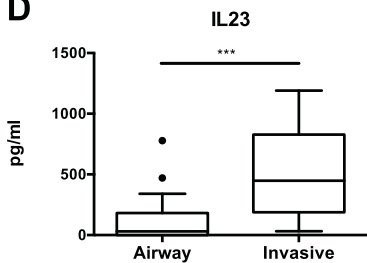**E**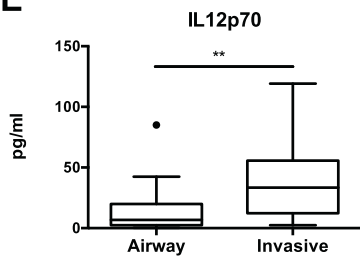**F**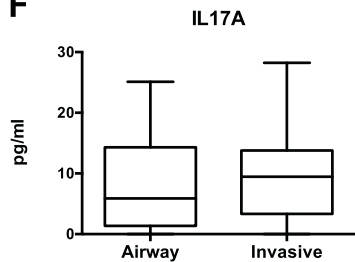**G**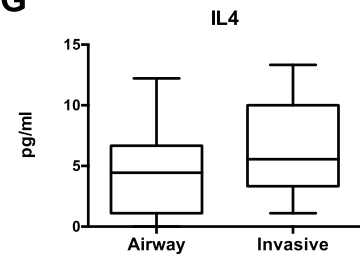

Supplement: Supplementary file 7 — Authors’ original file for figure 5 [file 12879_2013_3803_MOESM7_ESM.pdf]

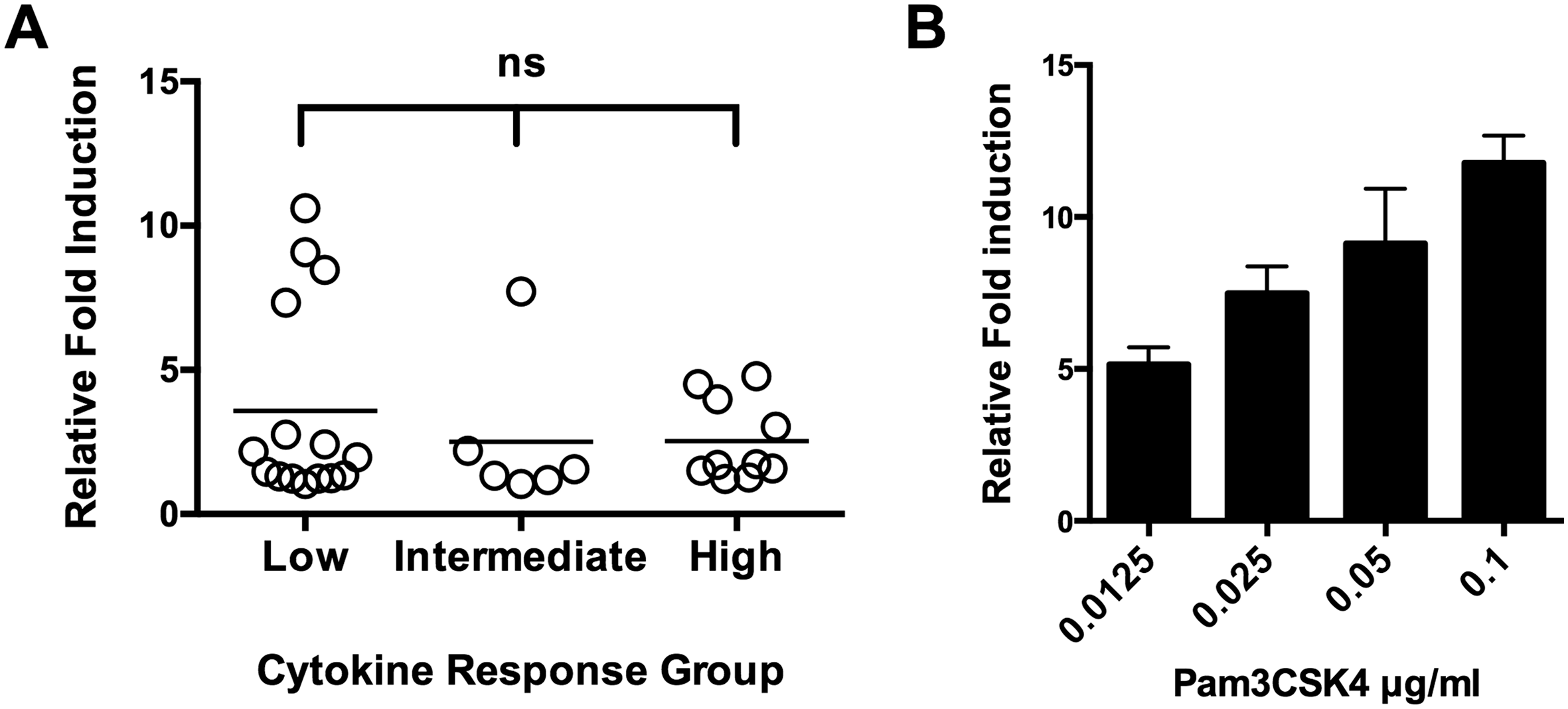

Supplement: Supplementary file 8 — Authors’ original file for figure 6 [file 12879_2013_3803_MOESM8_ESM.tif]
